# Observations on the symbiotic relationship between the caridean shrimp *Odontonia sibogae* (Bruce, 1972) and its ascidian host *Herdmania momus *(Savigny, 1816)

**DOI:** 10.1371/journal.pone.0192045

**Published:** 2018-02-21

**Authors:** Ya'arit Levitt-Barmats, Noa Shenkar

**Affiliations:** 1 School of Zoology, George S. Wise Faculty of Life Sciences, Tel Aviv University, Tel Aviv, Israel; 2 The Steinhardt Museum of Natural History, Israel National Center for Biodiversity Studies, Tel Aviv University, Tel Aviv, Israel; National Taiwan Ocean University, TAIWAN

## Abstract

Symbiotic relationships between shrimps and other invertebrates are a very common phenomenon in tropical environments. Although the caridean shrimp-ascidian association has been known for many years, the nature of this relationship is still unclear. The current study investigated the association between the caridean shrimp *Odontonia sibogae* (Bruce, 1972) and solitary ascidians. A combination of field work conducted along the Red Sea coast of Israel and laboratory experiments, conducted during 2015–2016, revealed a clear preference of the shrimps for the ascidian species *Herdmania momus* (Savigny, 1816), with a low survival ability of the shrimp outside their host's body. The shrimps usually inhabit their host as pairs of male and female or pair of females, but never as pairs of males. Out of the 53 studied females, 51% were observed to bear between 156–1,146 embryos, throughout the course of the year. As these ascidian hosts are known to create large aggregates, we suggest that males may possibly wander among the ascidians occupied by females in order to increase their reproductive success. To date, this is the first study to record the shrimp *Dactylonia ascidicola* (Borradaile, 1898) inhabiting the ascidian *H*. *momus*; and the first study to investigate in depth the ascidian-shrimp association in the Red Sea. It thus provides a platform for future research into the physiological and behavioral adaptations required for such a unique association.

## Introduction

Symbiotic relationships, defined as different organisms living together, usually involve small organisms that interact with larger hosts, with varied costs and benefits between the partners. Tropical coral reefs are considered to be one of the most complex marine environments, with a wide variety of symbiotic relationships [1–7].

Crustaceans can be found in diverse environments, from fresh-water to the deep sea, mostly as free-living organisms [8]. Other crustaceans, in particular shrimps, are found in various symbiotic relationships with numerous marine organisms. Moreover, symbiosis is probably one of the most common environmental adaptations in marine crustaceans, documented in numerous studies [2–4,9,10]. Some symbiotic species inhabit their host as a solitary dweller and defend their habitat against intruders, while others can be found in pairs or even in groups of three and more. In many cases, one large host can be inhabited by different symbiont groups, such as shrimps, crabs, ascidians and even fishs. Presumably, following settlement upon its host, the symbiont will not leave it unless forced to [2,3,11–14].

The host can provide its associate with a refuge and protection from predators, and some symbiont species even feed directly upon their host. Parasites of the subclass Branchiura suck blood or tissue fluids from their fish and amphibian hosts [2]. In contrast, some shrimp species are considered as cleaners of certain reef-fish species, controlling the fishes' gill, oral and external parasites [14–16]. Species of the genus *Periclimenes* living with sea anemones feed from their host's tentacles [17,18]. In many filter-feeder hosts, the endo-symbiont may feed on the host’s mucus with its entrapped detritus and on various particles pumped in for the host, as an energy-rich food supply [6]. Pea crabs feed on the food accumulated on the bivalve gill mucus [19]. The ecto-symbiont *Cuapetes tenuipes* (Borradaile, 1898) was also observed feeding on its coral host's mucus [20]. *Athanas* symbiont species living with sea urchin hosts have been observed consuming algal fragments, similar to their host [21]. Crabs living in the rectum of their sea urchin hosts were found to feed on the urchins' living tissues and feces [22]. Other crabs have been observed near or inside the cloacal cavities of several species of holothuroids, without harming to their hosts [23,24].

The Palaemonidae (Infraorder: Caridea) is a highly diverse Indo-Pacific family, with numerous symbiotic species, in particular with other invertebrates. While some of the shrimps display restriction to a single host species, others can be found with a variety of invertebrate groups. The endosymbiont species exhibit morphological adaptions such as a stout, swollen and flattened body, reduction or loss of the rostrum, reduced eyes, smoother carapace and abdomen, and sturdy clinging pereiopods to improve the movement on and inside their host. In addition, the majority of shrimp species display a color resemblance pattern to their host or have simply become transparent, to blend in with their host's body [4,6,25,26].

To date, only a few laboratory experiments have investigated the symbiotic behavior of caridean shrimps and their hosts. The shrimp *Periclimenes soror* Nobili, 1904 was found to actively orient to its cushion star host by means of chemical cues [27]. The nutritional need of *P*. *brevicarpalis* (Schenkel, 1902) in its sea anemone host was experimentally observed through the presence of the bubble-tip sea anemone *Entacmaea quadricolor* (Leuckart 1828) [17]. The mating system and other symbiont parameters of different shrimp species and their hosts have also been studied [12,28,29]. The first observation of a shrimp associated with an ascidian was published in 1910 [25]. Since then, ascidian-palaemonid shrimp associations have been observed, but laboratory studies have remained rare [9,13,26].

The current study site, the Red Sea, is well-known for its extraordinarily high biodiversity and high number of endemic species [30–33]. Palaemonid species are very common inhabitants of these coral reefs, with the majority of the symbiosis observations having been on stony coral, sea anemone, sea cucumber and sponge hosts, and mostly in the center and southern parts of the Red Sea [9,13,20,25,34,35]. In 2005–2006, during a study conducted along the coast of Eilat, Israel, 115 specimens of the solitary ascidian *Herdmania momus* (Savigny, 1816) were collected, particularly from artificial substrata. In 14% of these ascidians, the associated shrimp *Odontonia sibogae* (Bruce, 1972) were found [36]. *O*. *sibogae* were previously known from the Indian Ocean, Indonesia, Australia and New Caledonia, and have been found in association with the solitary ascidians *Styela whiteleggei* (Herdman, 1899), *H*. *momus*, *Rhopalaea crassa* (Herdman, 1880), and *Polycarpa* sp. In addition, there are some records of a few individuals collected from a coral bottom or under coral rocks and boulders [25].

The current study sought to investigate the association between *O*. *sibogae* and its ascidian hosts in the Red Sea, and to study the shrimp’s behavior with and without the host. The findings contribute to an understating of the necessity of this association for the shrimp, and whether there exists a preference for a specific ascidian host species.

## Materials and methods

### Hosts and shrimps collection

A total of 183 individuals of the solitary ascidian *Herdmania momus* were collected during 2015–2016, using SCUBA, snorkeling, or pulling ropes (Eilat Marina), at seven different locations, along the Red Sea coast of Israel (Table 1), with a total of 105 associated shrimps found inhabiting the ascidians (collection permit numbers 40764–41258). Randomly collected *H*. *momus* individuals were immediately placed in Ziploc bags or a 1 liter jar and transported to the Inter-University Institute for Marine Sciences (IUI) in Eilat. The collected organisms were kept in the same open water-table system where the experiments were carried out, with running sea-water (5uM pre-filtered), and without supplementary food. The number of shrimps per individual ascidian host was determined for each experiment as detailed below. In addition, the length of associated ascidians was measured (from the oral siphon to the base). Upon completion of each experiment the associated shrimps were preserved in 70% ethanol and vouchered in the Crustacea collection at the Steinhardt Museum of Natural History, Israel National Center for Biodiversity Studies at Tel-Aviv University. In the lab, using a Canon SMZ18 stereomicroscope, the identification of each shrimp was verified, and its sex was determined based on the endopod of the second pleopods, with their short appendices masculinae, which are present only in males [25]. Sex determination could not be determined for 14 shrimp individuals due to damage to critical body parts. Size was expressed as post-orbital carapace length (pocl), measured with a digital caliper to the nearest 0.1 mm, comprising the distance from the posterior orbital edge to the mid-dorsal posterior border of the carapace. The number of embryos in the ovigerous-females was counted.

**Table 1:** Collection sites and dates of the ascidian *Herdmania momus* hosting the caridean shrimp *Odontonia sibogae* along the Red Sea coast of Eilat, Israel.

Site	Coordinates	Depth (m)	Date	# of ascidians (shrimps)
Kisuski Water sports jetties	29°32'51 N 34°57'14 E	0.5	18/06/2015	1 (1)
			18/08/2015	10 (14)
			19/08/2015	19 (16)
			23/08/2015	13 (6)
			31/08/2015	7 (2)
			17/05/2016	8 (4)
			05/06/2016	29 (14)
			27/06/2016	9 (4)
			10/08/2016	6 (4)
North Beach- Sea Scouts platform	29°32'55 N 34°57'41 E	0.5	17/08/2015	14 (14)
North Beach- Floating Jetties	29°32'53 N 34°57'43 E	0.5	17/08/2015	1 (1)
Kisuski Water sports	29°32'50 N 34°57'14 E	5	18/08/2015	1 (1)
Eilat Marina	29°33'10 N 34°57'36 E		27/12/2015	1 (1)
			16/02/2016	21 (6)
		0.5	10/03/2016	1 (2)
			03/2016	10 (4)
			15/05/2016	7 (1)
			24/08/2016	6 (0)
Artificial coral Nursery	29°32'31 N 34°58'24 E	15	09/05/2016	9 (4)
			17/05/2016^a^	9 (6)
Sun Boat	29°32'42 N 34°58'70 E	12	04/07/2016	1 (1)

^a^ The collection site and date of the ascidians *Pyura gangelion* (Savigny, 1816) and *Phallusia nigra* Savigny, 1816 for the preference experiments.

### *Odontonia sibogae* survivorship experiment

**Fig 1:**
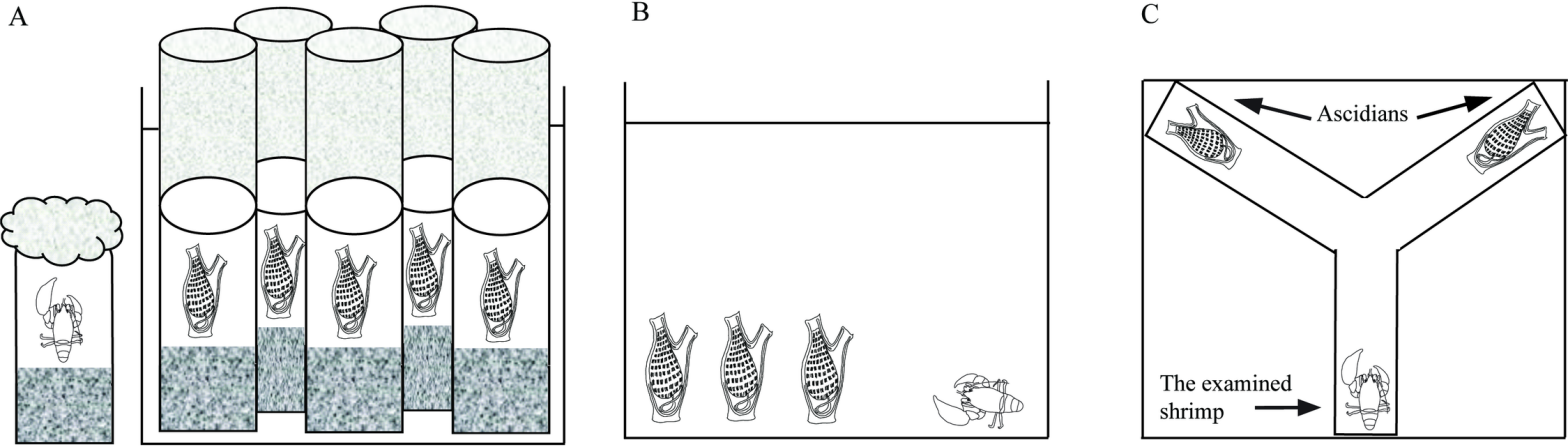
An illustration of the experimental arrangement at the Inter-University Institute for Marine Sciences in Eilat. (A) The survivorship experiment: the shrimp *Odontonia sibogae* in a separate net-covered cup, and the ascidian *Herdmania momus* in cups with stony bottoms and a long net sleeve reaching above the water level. (B) The preference experiment: the shrimp *O*. *sibogae* and the three-ascidian species in the aquarium. (C) The Y-maze with the shrimp *O*. *sibogae* at the base of the maze, and the two ascidian species placed at either side of the maze, used in the third trial.

Since we did not observe any free-living specimens of *Odontonia sibogae* in the natural coral reef in our field work, we hypothesized that the ascidian-shrimp association is obligatory. In order to test *O*. *sibogae*’s ability to survive outside the host's body, 66 individuals of the ascidian *H*. *momus* were collected, hosting 49 *O*. *sibogae* shrimp individuals in total, during August 2015. As we were not able to detect shrimp presence within the living host, approximately half of the collected ascidians (*n* = 30) were dissected. The separated shrimps (*n* = 26) were kept in individual cups with a long net sleeve cover in order to prevent them from wandering out (Fig 1A). In addition, 36 live ascidians were monitored, each in its own cup with a stony bottom and a long net sleeve, in order to investigate the absence/presence of a shrimp later on (Fig 1A). To ensure better water circulation, the ascidians were kept in smaller aquaria (30 X 25 X 30 cm) within the water-table. When one of the ascidians died, it was examined for the presence of shrimp symbionts. If such shrimps were alive, they were also kept in individual cups with a net covering. As long as the ascidians remained alive, no dead shrimp symbionts were detected. Survival was measured in number of days from the symbiont being separated from its host until it died. Temperature in the water-table was 26–27°C. Both ascidians and shrimps were examined every few hours for a period of one month. Since we were not able to maintain live ascidians for the full duration of the experiment we could not perform statistical analysis on these results.

### Preference experiments

Though *O*. *sibogae* have been documented as hosted by other ascidian species [25], along the Gulf of Aqaba coast they were found inhabiting only *H*. *momus* individuals. In order to ascertain whether the *O*. *sibogae*-*H*. *momus* symbiosis is species-specific, 33 individuals of the ascidian were collected during May 2016, hosting 12 *O*. *sibogae* shrimp individuals. In addition, three and five individuals, respectively, of the solitary ascidians *Pyura gangelion* (Savigny, 1816) and *Phallusia nigra* Savigny, 1816, were collected (Table 1). The shrimps were detached from their original hosts and kept in separate aquaria (17 X 28 X 20 cm), while the ascidians were kept in cups with a stony bottom, 3–5 cups in one aquarium (Fig 1B). Each aquarium was connected to the running seawater system of the open water-table by a tube. A net covering the water exit hole of each aquarium prevented the shrimps from escaping. The temperature in the water-table was kept constant at 24–25°C. As the following experiments were the first to follow *O*. *sibogae* behavior under laboratory conditions, we conducted two trials.

Trial I: Three species of ascidians and one rock were placed in the aquarium. The ascidian species were *H*. *momus* and *P*. *gangelion*, with *P*. *nigra* as control, since the latter is not known from the literature as a host of *O*. *sibogae*. After 10–15 minutes to allow the ascidians to become relaxed and open their siphons, one individual of the shrimp *O*. *sibogae* was released into the aquarium from above or into the opposite side and its behavior was observed for not less than 30 minutes or until it had entered one of the ascidian species (e.g., S1 Movie Clip). Once a shrimp had entered an ascidian neither were retested. The experiment was repeated 17 times, with ten different shrimp individuals.

Trial II: In order to determine whether *O*. *sibogae* use chemical cues to locate the host, the ascidian had to be concealed from the shrimp’s sight. One individual of each of the ascidians *Pyura gangelion* and *H*. *momus* were placed at either side of a Y-maze (Fig 1C). Both arms of the Y-maze were 20 cm long and the base was 23 cm long. The Y-maze was 5 cm wide and 11.5 cm deep. The maze was filled with fresh water from the running sea-water system (8 cm high) and was replaced after each two tests. After 10–15 minutes to allow the ascidians to become relaxed and open their siphons, one individual of the shrimp *O*. *sibogae* was released into the same location at the base of the Y-maze and its behavior was observed for 60 minutes or until it had entered one of the ascidian species. After five repeated tests with different shrimp individuals the location of the ascidian species was switched to the other arm of the Y-maze to neutralize the bias of a particular arm selection by the shrimp. This was followed by testing six additional shrimp individuals (total of 11 individuals).

### Statistical analysis

A paired *t* -test was used to determine any differences between carapace length (CL) in associated male-female and female-ovigerous female with CL as the dependent variable, and a chi-square test to detect any differences in dispersal location of the shrimps within their ascidian hosts, with the shrimp sex as the dependent variable. To determine whether a correlation exists between host size and symbiont size (measured as CL), a General Linear Model test was used (with 0.95 confidence interval), with CL as the dependent variable. A Mann-Whitney U test was used in order to uncover differences in the surviving shrimps, between shrimp size (measured as CL) and shrimp sex, with number of shrimp survival days as the dependent variable. Another chi-square test was used to detect the shrimp preference for an ascidian species, with shrimp choice behavior as the dependent variable. Lilliefors and Kolmogorov–Smirnov tests of normality were used for all studied data. All statistical tests were conducted using Statistica^TM^ package (StatSoft, Inc.Ver.8), and R version 3.2.0 (R Development Core Team, 2014).

## Results

### Taxonomic identification

Description of *Odontonia sibogae*: subcylindrical body, smooth carapace and abdomen. Rostrum reaching distal end of antennular peduncle. Telson with 5–6 pairs of submarginal dorsal spines at regular distances, posterior margin with three pairs of spines, lateral spines small. Antenula with peduncle and flagella short, with acutely produced distolateral tooth reaching distal margin of intermediate segment. Stylocerite short, about half length of basal segment. First pereiopods rather slender, second pereiopods similar in structure but unequal in size. Coloration of males and females in generally similar though the males seem to be yellower and the females pinker. This closely corresponds to the description in Fransen (2002)^[^^25^^]^ for the species, and also distinguishes the species from other *Odontonia* species.

*Herdmania momus* was identified following Rius and Shenkar (2012)^[^^37^^]^.

### General characters

**Fig 2:**
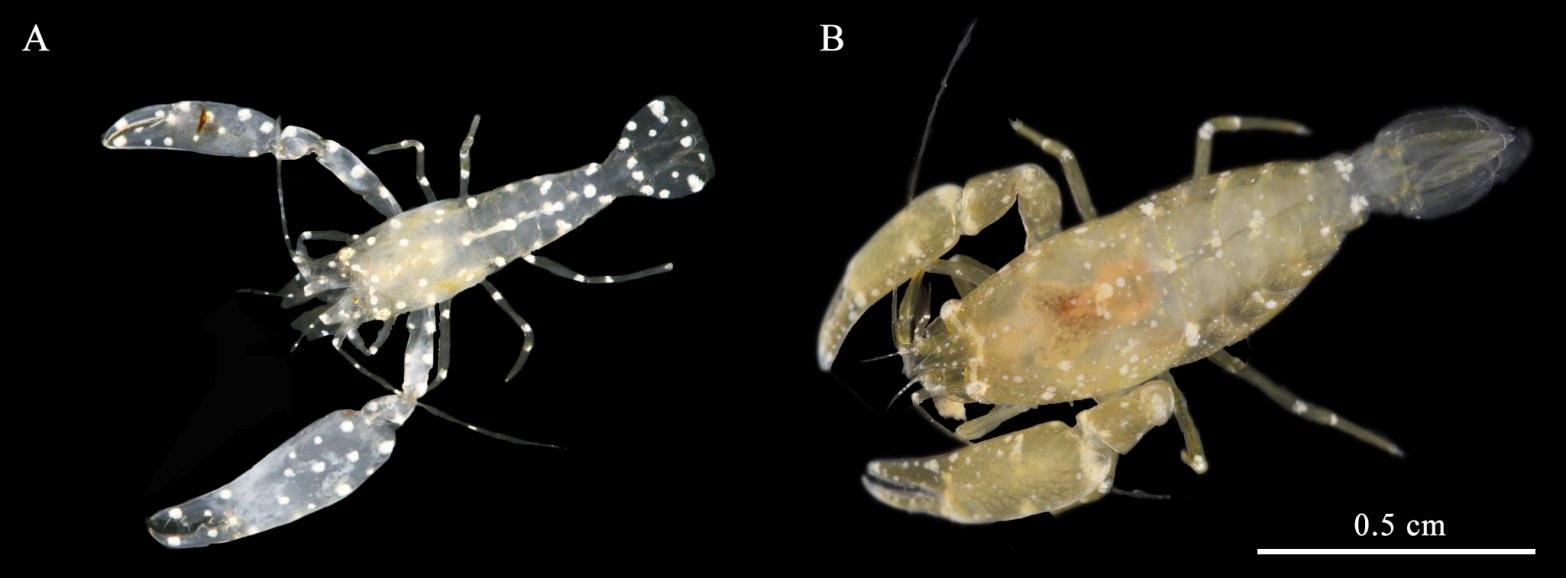
Associated caridean shrimps found within *Herdmania momus* along the coast of Eilat, Israel. (A) A male of *Dactylonia ascidicola*. (B) A female of *Odontonia sibogae*. Photograph: Rittner Oz.

**Fig 3:**
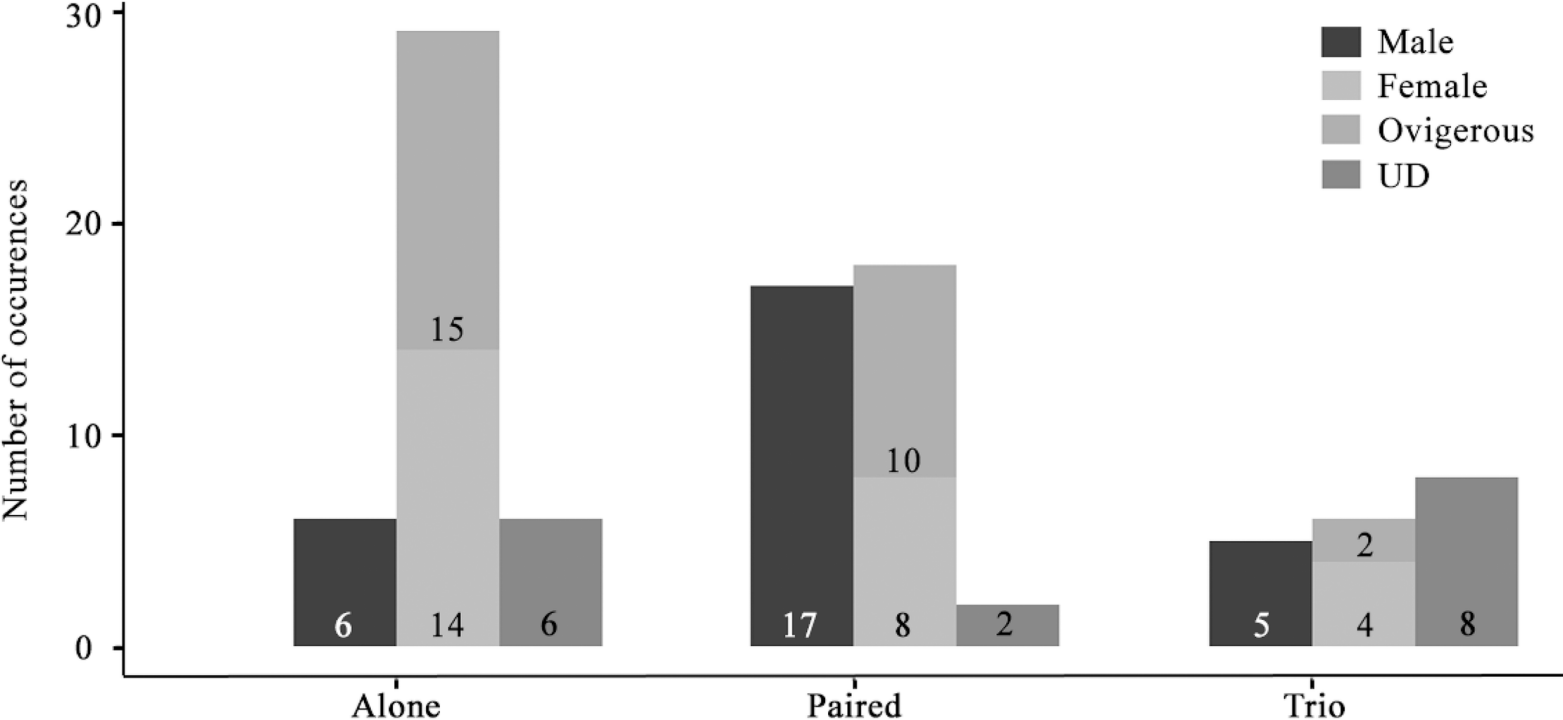
Number of occurrences of associations of the caridean shrimp *Odontonia sibogae* inhabiting *Herdmania momus*. Associations are characterized as: alone (single individuals), paired, trio, and UD (sex undetermined). Associations are separated by sex. Numbers in each column indicate the exact number of such combinations.

**Fig 4:**
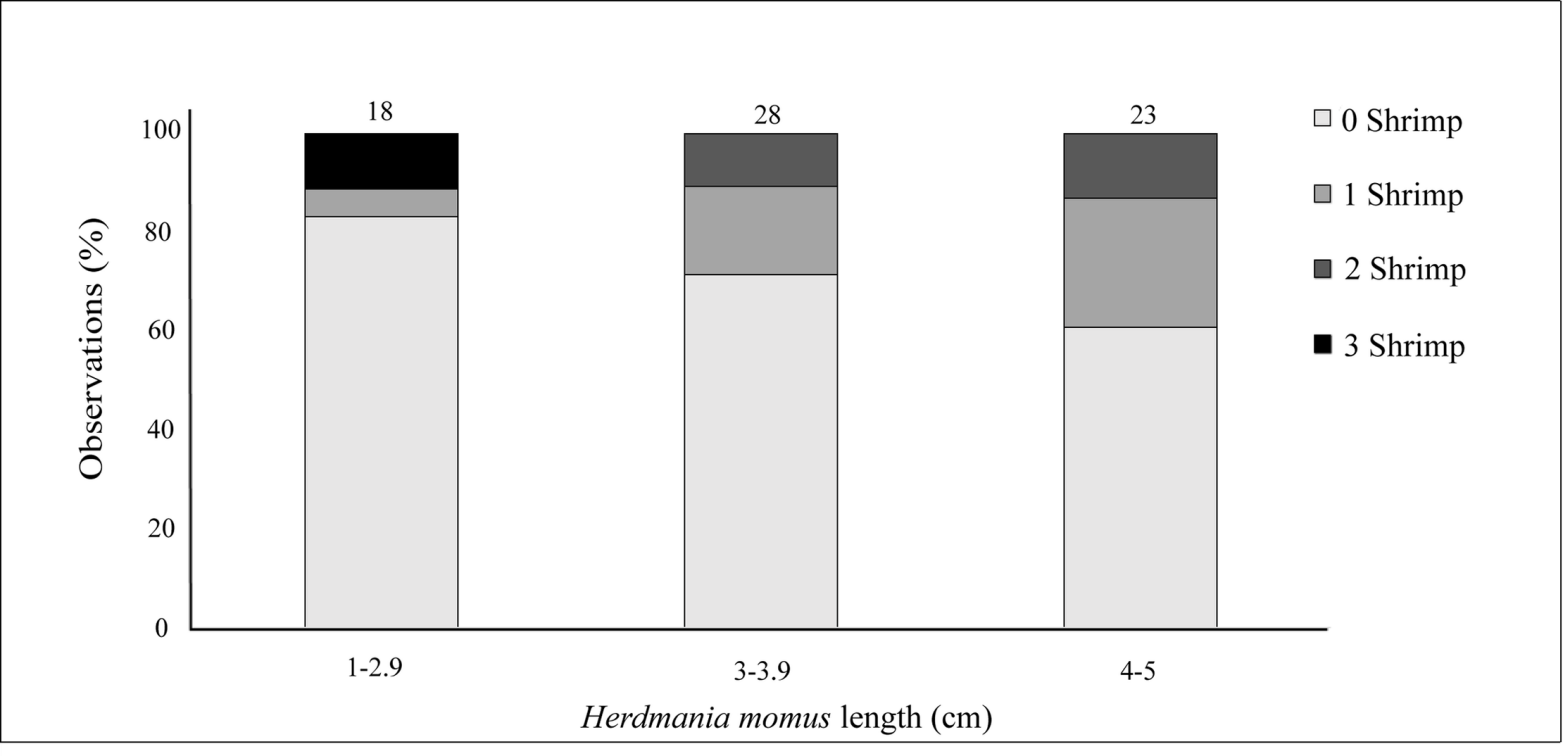
Relationship between *Herdmania momus* size and observations (percentage) of *Odontonia sibogae* individuals. The ascidian was measured from the oral siphon to the base (cm); color groups indicate zero, one, two or three individuals per individual host.

**Fig 5:**
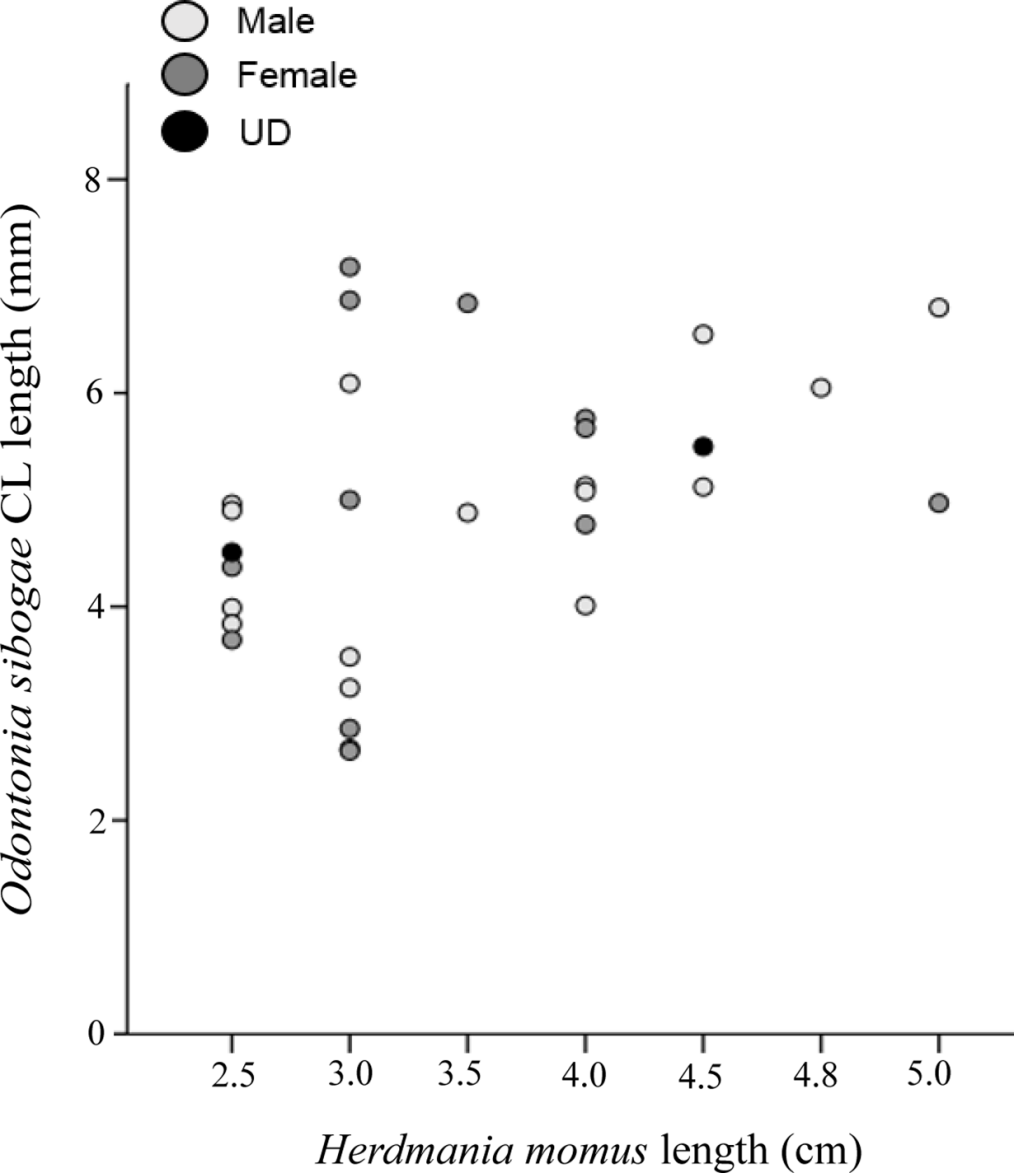
Relationship between host size and shrimp size. The ascidian was measured from the oral siphon to the base (cm), and the shrimp size was measured as carapace length (CL, mm) of females (dark gray circles) and males (bright gray circles); UD—sex undetermined (black circles).

Of the 183 *H*. *momus* individuals collected, 38.3% (*n* = 70) were infested with symbiotic shrimps. All shrimps were found behind the branchial sac, inhabiting the atrial chamber. The ascidian size ranged from1.5–5.1 cm (*n* = 83, 3.4 ± 0.9); however, shrimps were found only in ascidians larger than 2.5 cm (*n* = 74). Five *H*. *momus* individuals were found hosting eight individuals of the associated caridean shrimp *Dactylonia ascidicola* (Borradaile, 1898), a first record of this association (Fig 2A). The specimens fit the description of the species provided by Fransen (2002). In three of the associations, the shrimps were found in pairs of female and male, while the remaining *H*. *momus* were found with the caridean shrimp *O*. *sibogae* (*n* = 66, 36.1%) (Fig 2B). Twenty-seven associated ascidians hosted more than one *O*. *sibogae* shrimp (31.8%) and six ascidians were found with three *O*. *sibogae* individuals. More than 65% of the shrimps were female (*n* = 53) and half of these bore embryos (ovigerous, *n* = 27). The embryos were at different stages of development, 156–1,146 per female (average of 534.6 ± 374.7; *n* = 7). The average carapace length of female and male was 5.6 ± 1.2 (*n* = 24) and 5.5 ± 1.0 (*n* = 47) mm, respectively (Paired t test, *t*_*69*_ = -0.326, *P* = 0.746). Among the females the ovigerous individuals had an average carapace length of 5.9 ± 0.9 (*n* = 25). Though not statistically significant (Paired t test, *t*_*45*_ = -1.815, *P* = 0.0762), the ovigerous females were slightly larger than the non-ovigerous ones. Observation of distribution of the shrimps within the ascidian host individuals revealed that more males were found in male-female pairs than alone (Chi-square test, χ^2^ = 8.69, *P* < 0.01) (Fig 3). Despite the relatively low sample numbers, a positive trend was observed between the ascidian size class and the number of associations, with the largest ascidian size class (4–5 cm in diameter) characterized by the highest percentage of individuals that hosted shrimps (*n* = 9, 39%) (Fig 4). The correlation between host size and its shrimp symbiont size was not statistically significant (General Leaner Model test, *F*_2, 13_ = 0.88, *P* = 0.44)(Fig 5).

### *Odontonia sibogae* survivorship experiment

Presumably as a result of problems with the water circulation in the system, most of the ascidians did not survive more than a week, with only 11 individuals surviving more than two weeks. All ascidians died within 20 days. All the symbiont shrimps survived along with their live hosts, but after an ascidian died most of the shrimps were found clinging to their host tunicate. We separated some of these from their host, while those that were not separated died together with the ascidian.

**Fig 6:**
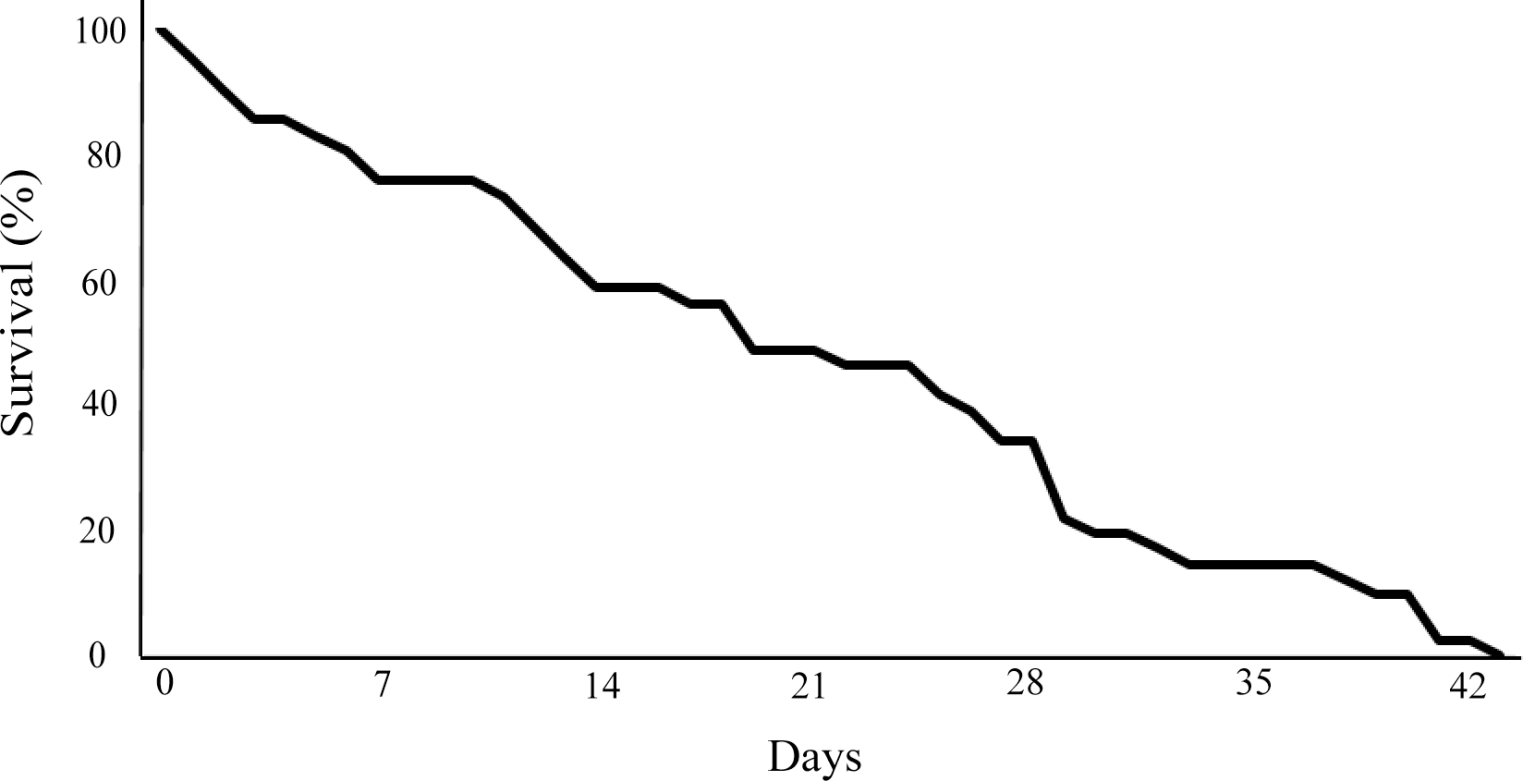
Survival curve of *Odontonia sibogae* separated from their host (days).

Twenty days post death of the hosts, less than 50% of the separated *O*. *sibogae* individuals still survived (*n* = 20), and less than 20% by day 31 (*n* = 8; Fig 6). No significant differences were found between males and females or in carapace length among the surviving shrimps (Mann-Whitney U test, U = 83.5, N_1_ = 23, N_2_ = 8, *P* = 0.72; U = 6, N_1_ = 7, N_2_ = 3, *P* = 0.36, respectively).

### Preference experiments

Numerous field observations have documented *O*. *sibogae* individuals in symbiosis only with *H*. *momus* species. Indeed, during the laboratory trials the shrimps entered only *H*. *momus* (three times during the aquarium experiment and three times in the Y-maze experiment), mostly through the atrial siphon. However, we encountered difficulty in studying the shrimp behavior because they only showed a response to the stimulation in half of the trials (20 out of 41 trials). In the aquarium experiment presenting a choice of three ascidian species, the shrimps chose to sit once on the *Pyura gangelion* species and once on the *Phallusia nigra* species, while choosing *H*. *momus* five times. In the Y-maze the shrimps chose *H*. *momus* four times and *P*. *gangelion* three times out of the 11 tests of this trial. In all cases when *O*. *sibogae* chose *P*. *gangelion* it had turned towards the right arm of the maze, but did not enter the ascidian. Notwithstanding, no statistical significance was found in regard to the shrimps displaying a choice behavior or not; or when it did, in regard to which ascidian species it chose (Chi-square test, χ^2^ = 1.35, *P* = 0.245; χ^2^ = 2.54, *P* = 0.28 respectively).

In total, the shrimps displayed a choice behavior in seven out of the 17 experiments in the aquarium trial, and in seven of the 11 experiments in the Y- maze trial. In 43% of cases when the shrimp exhibited a choice behavior it eventually entered the ascidian.

## Discussion

The Red Sea is well known for its high biodiversity and unique interactions among organisms [25,31]. Here we documented the unique association between the solitary ascidian *Herdmania momus* and the symbiotic shrimp *Odontonia sibogae*. This association was found to be more common than previously assumed, and appears crucial for *O*. *sibogae* survival.

The current study documents a highly common interaction between *O*. *sibogae* and the solitary ascidian *H*. *momus*. As ovigerous-females were found in most of the samples taken throughout the year, it seems that they reproduce year-round, similar to other shrimp species in tropical regions [38,39]. It is fairly common to find symbiont shrimp sharing the same coral colony with other symbionts such as small fishs and other crustacean species, as the coral complexity provides sufficient area for different organisms to share this habitat. However, because ascidians are smaller and provide fewer symbiotic niches, this is probably why different decapod species are not found cohabiting within one ascidian individual.

Knowlton (1980)^[^^11^^]^ suggested that the males shrimp do not necessarily remain for long periods of time with one sea anemone host but may wander from female to female in order to increase their reproductive effort, and also to defend their female mate against competitive males. In the current study we found fewer males than females inhabiting the ascidian hosts, and in most cases males were found only in the presence of another female and occasionally with a female and juvenile. Interestingly, two males were never found together within one ascidian host. In addition, though the differences in size between males and females were small, no correlation was found between the host and the associated shrimp body size. Our study thus supports Knowlton’s hypothesis, together with findings from other recent studies on other symbiont crustaceans [7,12]. *Herdmania momus*’s preferred habitat is on artificial substrata, and in Eilat they are commonly found in aggregations and / or near other *H*. *momus* individuals, and can grow in large densities on floating docks, displaying a fast growth rate [40]. The presence of adjacent ascidian host individuals in great numbers can contribute to the male shrimp’s reproductive success by enabling it to wander among the females within the *H*. *momus* host individuals.

To date, the symbiont shrimp *Dactylonia ascidicola* has been found in association with ‘unknown’ black ascidians (original description) and also with *Ascidia* species, as well as *Rhopalaea crassa* (Herdman, 1880) [25]. Along the coast of Eilat it is found in particular in association with the phlebobranch ascidian *Phallusia nigra* (Levitt-Barmats unpubl data). Due to the lack of accurate taxonomic identification of the ascidian host in previous publications, we cannot determine whether the occurrence of *D*. *ascidicola* with *P*. *nigra* is also a first record.

### *Odontonia sibogae* survivorship experiment

*H*. *momus* can flourish and establish massive populations without the presence of *O*. *sibogae*, as introduced populations of this species are commonly found nowadays in the Mediterranean without any associated shrimp [36,41]. In contrast, even though our experimental *O*. *sibogae* were kept in running sea water and protected from predators, they did not survive for long outside their host. Nonetheless, some food-searching behavior was observed during the experiments, in addition to the reports in the literature of field observations of *O*. *sibogae* outside a host. During the experiments, some of the separated shrimps were observed positioned against the running water tube and spreading their chelae as if trying to catch particles flushing into the aquarium. In another case, the wandering shrimps were seen moving around in the aquarium with their opened chelae during the night hours, but there were no observations of wandering shrimps in the presence of an ascidian host. In addition, observation of four *O*. *sibogae* individuals from the Eilat population, kept at Tel Aviv University in three separate aquaria (one male-female pair in one aquarium and two additional ovigerous-females in the other two), revealed that in the presence of *H*. *momus* individuals from the Mediterranean Sea, all four of these shrimps entered into the ascidians during the night.

The ascidian atrial chamber, which the symbiotic shrimp seem to occupy, receives a high-water circulation as a result of the ascidian’s filtration. This water includes the ascidian feces and post-filtration matter. The shrimp can also approach the ascidian’s gonads, which offer a protein-rich supplement for the symbiont. From this location, however, it cannot reach the host’s mucus or the inflow particles without tearing the ascidian branchial sac and thereby probably severely damaging its host.

### Preference experiments

The laboratory experiments carried out here revealed a species-specific association that follows the field observations documenting *O*. *sibogae* in the Red Sea as found only in *H*. *momus*, though in the literature there are observations of its symbiosis with additional ascidian species. A similar behavior was observed for the snapping shrimp *Alpheus roquensis* Knowlton & Keller, 1985, although it occurs mostly in association with the sea anemone *Ragactis lucida* (Duchassaing and Michelotti 1860), but has also occasionally been observed with the sea anemone *Bartholomea annulata* (Le Sueur, 1817) in areas where *R*. *lucida* was not abundant [42,43]. The alpheid shrimp *Arete indicus* Coutière, 1903, is considered a specific-species symbiont, as in both field and laboratory observations it preferred the sea urchin *Echinometra mathaei* (Blainville, 1825), and was only once documented in the field with another sea urchin species [21]. Some caridean species are also known to occupy gastropod shells such as *Pontonia chimaera* Holthuis, 1951, which is the only known palaemonid-gastropod association, and *Aretopsis amabilis* De Man, 1910, which is associated with hermit crabs [25,44,45]. In a similar set of laboratory experiments carried out on *Periclimenes* species with their sea anemone hosts, the shrimp *Periclimenes ornatus* Bruce, 1969 also displayed a preference for the same sea anemone species with which it was commonly observed in the field, compared to the other examined *Periclimenes* species that had demonstrated a more variable host preference [46]. Laboratory observations investigating distribution patterns of the symbiotic crab *Tetralia rubridactyla* Garth, 1971 revealed that host species-specificity contributed less to the crab preference than coral abundance and the territorial behavior of the crab itself [47]. This may explain *O*. *sibogae*’s presence with other ascidian host species in areas where *H*. *momus* is less abundant. *Ascidonia californiensis* (Rathbun, 1902), though known only from the California area in the United States, was observed inhabiting two ascidian species of the genus *Ascidia*. *Pontonia pinnophylax* (Otto, 1821), which is found in association with bivalves, mostly of pinnid species, demonstrates different species preferences depending on its geographical habitat [25]. *Lysmata grabhami* (Gordon, 1935) and *Lysmata seticaudata* (Risso, 1816) also provide examples of sea anemone symbiont shrimp that are not species-specific and can be found without a host at all, but inside cracks and crevices [14]. It thus seems that in more generalist shrimp symbiont species, including partly free-living symbionts, which can be associated with several different hosts, the shrimp will still choose to occupy organisms belonging to the same systematic group [13].

Previous laboratory experiments on the ability of symbiotic shrimps to locate their host non-visually have shown that the shrimp employ chemical cues to orient to the preferred host species (e.g. sea anemones or cushion star hosts; [27,46]). Here we could not determine whether *O*. *sibogae* employ any kind of chemical cue to identify their host, due to the relatively small volume of water in which they were kept. Future experiments in the field may contribute to our understanding of how *O*. *sibogae* locate their host in the highly diverse and complex reef system.

## Conclusions

It appears that *Odontonia sibogae* shrimp cannot survive for long outside their host and that they have a preference for a specific ascidian species, as, when given a choice, they entered only *Herdmania momus*. Although it is unlikely that the ascidian benefits from this association, it is still unclear as to whether the shrimps harm their host or constitute commensal symbionts, as they may cause some reduction in the hosts’ growth or in their reproductive ability [6]. Future examination of the shrimp’s digestive system content could provide us with additional information regarding its diet. Inside their hosts, the shrimp can enjoy a safer environment from predators and the ovigerous-females can remain much more protected. The dense aggregations of *H*. *momus* assist *O*. *sibogae* males to fertilize a larger number of females and thus increase their reproductive success.

The current study has provided an in-depth investigation of an overlooked, yet relatively common, symbiotic association. The evolutionary adaptation for this association demonstrated through the shrimp’s specific morphology and body color adaptation, combined with its selective behavior, serves as a basis for future research into such aspects as diet preferences or reproductive behavior, and for investigating the ability of the associated shrimp to expand its geographic distribution as a "hitch-hiker" within its host.

## Supporting information

S1 Movie Clip A movie of a female *Odontonia sibogae* entering the ascidian *Herdmania momus*. (MP4)

S1 Datafile Sex and individual length per each collected shrimp. (XLSX)

## References

[1] Leung TLF, Poulin R. Parasitism, commensalism, and mutualism: Exploring the many shades of symbioses. Vie Milieu. 2008;58: 107–115.

[2] Baeza JA. Crustaceans as Symbionts: An Overview of Their Diversity, Host Use, and Lifestyles In: Thiel M, Watling L, editors. Lifestyles and Feeding Biology The Natural History of the Crustacea, Vol 2. Oxford: Oxford University Press; 2015 pp. 163–189.

[3] Thiel M, Baeza JA. Factors Affecting the Social Behaviour of Crustaceans Living Symbiotically with Other Marine Invertebrates: A Modelling Approach. Symbiosis. 2001;30: 163–190.

[4] Ross MD. Symbiotic relationships In: Bliss D, editor. The biology of Crustacea. New York: Academic Press; 1983 pp. 163–212. Available: https://books.google.co.il/books?hl=iw&lr=&id=Re_sBe-xQtQC&oi=fnd&pg=PA163&dq=Symbiotic+relationships.+In+The+biology+of+Crustacea&ots=KtE09bElwk&sig=7OaYIWYjaF4ZSNYqa4d0cPaCro8&redir_esc=y#v=onepage&q=Symbioticrelationships

[5] Harmelin-Vivien ML. Reef fish community structure: an Indo-Pacific comparison In: Harmelin-Vivien ML, François B, editors. Vertebrates in complex tropical systems. Berlin Heidelberg New York: Springer; 1989 pp. 21–60. Available: http://link.springer.com/chapter/10.1007/978-1-4612-3510-1_2#page-1

[6] Bauer RT. Remarkable shrimps: adaptations and natural history of the carideans (Vol. 7). University of Oklahoma Press: Norman; 2004.

[7] Baeza JA, Thiel M. The mating system of symbiotic crustaceans. A conceptual model based on optimality and ecological constraints In: Duffy JE, Thiel M, editors. Reproductive and Social Behavior: Crustaceans as Model Systems. Oxford: Oxford University Press; 2007 pp. 245–255. doi: 10.1093/acprof

[8] Ruppert EE, Fox RS, Barnes RD. Invertebrate Zoology 7th ed. California, USA: Brooks/Cole; 2004.

[9] Fransen CHJM. On Pontoniinae (Crustacea, Decapoda,Palaemonidae) collected from ascidians. Zoosystema. 2006;28: 713–746.

[10] Westinga EPHC, Hoetjes PC. The intrasponge fauna of *Spheciospongia vesparia* (Porifera, Demospongiae) at Curaçao and bonaire. Mar Biol. 1981;62: 139–150. doi: 10.1007/BF00388176

[11] Knowlton N. Sexual selection and dimorphism in two demes of a symbiotic, pair-bonding snapping shrimp. Evolution (N Y). 1980;34: 161–173. doi: 10.2307/2408325 10.1111/j.1558-5646.1980.tb04802.x 28563213

[12] Baeza JA, Hemphill CA, Ritson-Williams R. The sexual and mating system of the shrimp *Odontonia katoi* (Palaemonidae, Pontoniinae), a symbiotic guest of the ascidian *Polycarpa aurata* in the Coral Triangle. PLoS One. 2015;10: 1–18. doi: 10.1371/journal.pone.0121120 25799577 10.1371/journal.pone.0121120 PMC4370848

[13] Bruce AJ. Coral Reef Caridea and “Commensalism.” Micronesica. 1976;12: 83–98.

[14] Wirtz P. Crustacean symbionts of the sea anemone *Telmatactis cricoides* at Madeira and the Canary Islands. J Zool Soc London. 1997;242: 799–811. doi: 10.1111/j.1469-7998.1997.tb05827.x

[15] Limbaugh C, Pederson H, Chace FA. Shrimps that clean fishes. Bull Mar Sci Gulf Caribb. 1961; 237–257.

[16] Van Tassell JL, Brito A, Bortone SA. Cleaning Behavior among marine fishes and invertebrates in the Canary Islands. Cybium. 1994;18: 117–127.

[17] Fautin DG, Guo C-C, Hwang J-S. Costs and benefits of the symbiosis between the anemone shrimp *Periclimenes brevicarpalis* and its host *Entacmaea quadricolor*. Mar Biol. 1995;129: 77–84.

[18] Grippa GB, D’Udekem D’ Acoz C. The genus Periclimenes Costa, 1844 in the Mediterranean Sea and the Northeastern Atlantic Ocean: review of the species and description of Periclimenes sagittifer aegylios subsp. nov. (Crustacea, Decapoda, Caridea, Pontoniinae). Atti della Soc Ital di Sci Nat e del Mus Civ di Stor Nat di Milano. 1996;135: 401–412.

[19] Chaparro OR, Saldivia CL, Paschke KA. Regulatory aspects of the brood capacity of *Crepidula fecunda*, Gallardo 1979 (Gastropoda: Calyptraeidae). J Exp Mar Bio Ecol. 2001;266: 97–108. doi: 10.1016/S0022-0981(01)00336-7

[20] Bruce AJ, Svoboda A. Observations upon some pontoniine shrimps from Aqaba, Jordan. Zool Verh. 1983;205: 1–44.

[21] Gherardi F. Eco-ethological aspects of the symbiosis between the shrimp *Athanas indicus* (Coutiere 1903) and the sea urchin *Echinometra mathaei* (de Blainville 1825). Trop Zool. 1991;4: 107–128. doi: 10.1080/03946975.1991.10539481

[22] Castro P. Symbiosis between Echinoecus pentagonus (Crustacea, Brachyura) and its host in Hawaii, Echinothrix calamaris (Echinoidea). PhD. Thesis, University of Hawaii, Honolulu. 1969.

[23] Spiridonov VA. Results of the Rumphius Biohistorical Expedition to Ambon Part 8. Swimming crabs of Ambon (Crustacea: Decapoda: Portunidae). Zool Meded. 1999;73: 63–97. Available: http://www.repository.naturalis.nl/record/215086

[24] Vannini M, Innocenti G. Research on the coast of Somalia. Portunidae (Crustacea Brachyura). Trop Zool. 2000;13: 251–298.

[25] Fransen CHJM. Taxonomy, phylogeny, historical biogeography, and historical ecology of the genus *Pontonia* Latreille (Crustacea: Decapoda: Caridea: Palaemonidae). Zoologische Verhandelingen. 2002 doi: 10.1163/156854008784513483

[26] De Grave S. Biogeography of Indo-Pacific Pontoniinae (Crustacea, Decapoda): a PAE analysis. J Biogeogr. 2001;28: 1239–1253. doi: 10.1046/j.1365-2699.2001.00633.x

[27] Olliff ERR. Symbiosis of the sea star shrimp, *Periclimenes soror* Nobili, 1904 (Decapoda, Palaemonidae), and cushion star, *Culcita novaeguineae* Müller & Troschel, 1842 (Echinodermata, Asteroidea, Oreasteridae): host finding and benefits. Crustaceana. 2013;86: 564–577. doi: 10.1163/15685403-00003198

[28] Baeza JA, Bolaños JA, Hernandez JE, Lira C, López R. Monogamy does not last long in *Pontonia mexicana*, a symbiotic shrimp of the amber pen-shell *Pinna carnea* from the southeastern Caribbean Sea. J Exp Mar Bio Ecol. 2011;407: 41–47. doi: 10.1016/j.jembe.2011.07.011

[29] Baeza JA, Ritson-Williams R, Fuentes MS. Sexual and mating system in a caridean shrimp symbiotic with the winged pearl oyster in the Coral Triangle. J Zool. 2013;289: 172–181. doi: 10.1111/j.1469-7998.2012.00974.x

[30] Goren M. Statistical aspects of the Red Sea ichthyofauna. Isr J Zool. 1993;39: 293–298.

[31] Hariri KI, Nichols P, Krupp F, Mishrigi S, Barrania A, Ali FA, et al. Status of the Living Marine Resources in the Red Sea and Gulf of Aden and their Management. Strategic Action Programme for the red Sea and Gulf of Aden, final Report. Jeddah, Saudi Arabia; 2000.

[32] Brokovich E, Baranes A, Goren M. Habitat structure determines coral reef fish assemblages, Red Sea. Ecol Indic. 2006;6: 494–507. doi: 10.1016/j.ecolind.2005.07.002

[33] Dibattista JD, Roberts MB, Bouwmeester J, Bowen BW, Coker DJ, Lozano-Cortés DF, et al A review of contemporary patterns of endemism for shallow water reef fauna in the Red Sea. J Biogeogr. 2016;43: 423–439. doi: 10.1111/jbi.12649

[34] Vine P. Red Sea Invertebrates. London: IMMEL Publishing; 1986.

[35] Ďuriš Z. Palaemonid shrimps (Crustacea: Decapoda) of Saudi Arabia from the “Red Sea Biodiversity Survey” 2011–2013, with 11 new records for the Red Sea. Mar Biodivers. 2017; 1–15. doi: 10.1007/s12526-017-0681-8

[36] Shenkar N, Loya Y. The solitary ascidian *Herdmania momus*: native (Red Sea) versus non-indigenous (Mediterranean) populations. Biol Invasions. 2008;10: 1431–1439. doi: 10.1007/s10530-008-9217-2

[37] Rius M, Shenkar N. Ascidian introductions through the Suez Canal: The case study of an Indo-Pacific species. Mar Pollut Bull. Elsevier Ltd; 2012;64: 2060–2068. doi: 10.1016/j.marpolbul.2012.06.029 22857711 10.1016/j.marpolbul.2012.06.029

[38] Bauer RT. Continuous reproduction and episodic recruitment in nine shrimp species inhabiting a tropical seagrass meadow. J Exp Mar Bio Ecol. 1989;127: 175–187. doi: 10.1016/0022-0981(89)90183-4

[39] Bauer RT. Testing generalizations about latitudinal variation in reproduction and recruitment patterns with sicyoniid and caridean shrimp species. Invertebr Reprod Dev. 1992;22: 193–202. doi: 10.1080/07924259.1992.9672272

[40] Koplovitz G, Shmuel Y, Shenkar N, Bullard S. Floating docks in tropical environments—a reservoir for the opportunistic ascidian *Herdmania momus*. Manag Biol Invasions. 2016;7: 43–50. doi: 10.3391/mbi.2016.7.1.06

[41] Gewing M-T, Rothman SBS, Nagar LR, Shenkar N, Wong M. Early stages of establishment of the non-indigenous ascidian *Herdmania momus* (Savigny, 1816) in shallow and deep water environments on natural substrates in the Mediterranean Sea. BioInvasions Rec. 2014;3: 77–81. doi: 10.3391/bir.2014.3.2.04

[42] Hurt C, Silliman K, Anker A, Knowlton N. Ecological speciation in anemone-associated snapping shrimps (*Alpheus armatus* species complex). Mol Ecol. 2013;22: 4532–4548. doi: 10.1111/mec.12398 23859595 10.1111/mec.12398

[43] Knowlton N, Keller B. Two more sibling species of alpheid shrimps associated with the Caribbean Sea anemones *Bartholomea annulata* and *Heteractis lucida*. Bull Mar Sci. 1985;37: 893–904.

[44] Holthuis LB. A General Revision of the Palaemonidae (Crustacea Decapoda Natantia) of the Americas. I. the Subfamilies Euryrhynchinae and Pontoniinae. Allan Hancock Found Publ Occ Pap 1951;11: 1–332.

[45] Bruce AJ. *Aretopsis amabilis* de man: an alpheid shrimp commensal of pagurid crabs in the Seychelles Islands. Mar Biol Assoc India. 1969;11: 115–181.

[46] Guo C-C, Hwang J-S, Fautin DG. Host selection by shrimps symbiotic with sea anemones: A field survey and experimental laboratory analysis. J Exp Mar Bio Ecol. 1996;202: 165–176. doi: 10.1016/0022-0981(96)00020-2

[47] Limviriyakul P, Tseng LC, Shih TW, Hwang J-S. Host selection and preferences of coral symbiotic crab *Tetralia rubridactyla*. J Exp Mar Bio Ecol. Elsevier B.V.; 2016;485: 24–34. doi: 10.1016/j.jembe.2016.08.001

